# Helping in cooperatively breeding long-tailed tits: a test of Hamilton's rule

**DOI:** 10.1098/rstb.2013.0565

**Published:** 2014-05-19

**Authors:** Ben J. Hatchwell, Philippa R. Gullett, Mark J. Adams

**Affiliations:** Department of Animal and Plant Sciences, University of Sheffield, Sheffield S10 2TN, UK

**Keywords:** *Aegithalos caudatus*, altruism, inclusive fitness, kin selection, relatedness, social evolution

## Abstract

Inclusive fitness theory provides the conceptual framework for our current understanding of social evolution, and empirical studies suggest that kin selection is a critical process in the evolution of animal sociality. A key prediction of inclusive fitness theory is that altruistic behaviour evolves when the costs incurred by an altruist (*c*) are outweighed by the benefit to the recipient (*b*), weighted by the relatedness of altruist to recipient (*r*), i.e. Hamilton's rule *rb* > *c*. Despite its central importance in social evolution theory, there have been relatively few empirical tests of Hamilton's rule, and hardly any among cooperatively breeding vertebrates, leading some authors to question its utility. Here, we use data from a long-term study of cooperatively breeding long-tailed tits *Aegithalos caudatus* to examine whether helping behaviour satisfies Hamilton's condition for the evolution of altruism. We show that helpers are altruistic because they incur survival costs through the provision of alloparental care for offspring. However, they also accrue substantial benefits through increased survival of related breeders and offspring, and despite the low average relatedness of helpers to recipients, these benefits of helping outweigh the costs incurred. We conclude that Hamilton's rule for the evolution of altruistic helping behaviour is satisfied in this species.

## 1. Introduction

Our understanding of evolution has been transformed in the 50 years since Hamilton [1] published his seminal paper on inclusive fitness theory. Hamilton's insight that selection operates on genes that share common interests with copies of themselves carried by other individuals revolutionized the study of social evolution in its broadest sense [2–5]. Among its many consequences for the field of evolutionary biology, Hamilton's conceptual leap motivated the instigation of many long-term field studies of avian and mammalian cooperative breeding systems [6,7]. One of the earliest themes to emerge from these studies, leading to the early acceptance of the process of kin selection as a key driver of vertebrate sociality, was that cooperative breeding involving apparently altruistic care by non-breeders generally occurs within family groups. The factors promoting the formation of family groups have been extensively reviewed elsewhere [8–12], but regardless of the phylogenetic, ecological and life-history correlates of cooperation, relatedness is a very common (although not universal) feature of such systems [13,14]. This view has been reinforced by recent studies demonstrating that cooperative breeding systems are characterized by low promiscuity [15,16], a pattern that is also evident in the most complex and sophisticated societies, found among eusocial insects, that have evolved via a ‘monogamy window’ [17,18].

Despite the wide acceptance of the view that kin selection has played a major role in the evolution of avian cooperative breeding systems, the number of studies providing convincing empirical support for the predictions of inclusive fitness theory is more limited. Several investigations have shown that the relatedness between potential helpers and recipients influences the probability that helping occurs, both in observational [19,20] and experimental [21] studies. Similarly, the amount of care invested in a brood by helpers is associated with their relatedness to the recipients in several species [22–24]. Furthermore, meta-analyses support the contention that kin discrimination is a widespread trait among cooperatively breeding vertebrates [25,26]. These studies all imply a role for kin selection, but rather few studies have evaluated the relative importance of direct and indirect benefits in the evolution or maintenance of helping. Among the first attempts to do so were studies by Vehrencamp [27], Woolfenden & Fitzpatrick [28], Koenig & Mumme [29] and Russell & Rowley [30], reviewed by Emlen [31], that estimated the ‘index of kin selection’ [27]—the ratio of indirect fitness to inclusive fitness. However, the estimation of fitness components in these early studies often required assumptions about relatedness that, in retrospect, are invalid, and, in species with strongly age-structured life histories, assessment of alternative options for individuals is problematic [32,33]. More recent studies have attempted to quantify the direct and indirect components of inclusive fitness informed by genetic analysis, e.g. in Seychelles warblers *Acrocephalus sechellensis* [34] and long-tailed tits *Aegithalos caudatus* [35], and in the latter case, following Woolfenden & Fitzpatrick [28], fitness estimates were based on long-term measure of individuals’ lifetime reproductive success.

Despite the important advances made by these studies, most of which have strongly supported kin selection as a key process in avian social evolution, very few studies have attempted to directly test a key prediction of inclusive fitness theory, Hamilton's rule. Hamilton [1] argued that heritable social traits will be selected for when the cost of the social action (*c*) is outweighed by the indirect benefit, which is the product of the benefit to the recipient (*b*), weighted by the relatedness (*r*) of the recipient to the actor: *rb* > *c*. The scarcity of direct tests of Hamilton's rule does not only apply to social birds but is a more general problem. In a recent review, Bourke [36] identified 12 studies across all taxa that provide genetic and demographic data from natural populations that allow an explicit test of Hamilton's rule to be conducted, of which three involved vertebrates and just one a cooperatively breeding bird [37]. The aforementioned studies that quantified Vehrencamp's [27] index of kin selection, while offering important insights into the relative importance of kin selection, do not test Hamilton's rule *per se*. This relative paucity of evidence in support of a key prediction of inclusive fitness theory has led some authors to question the validity and utility of the theory itself [38].

**Figure 1.**
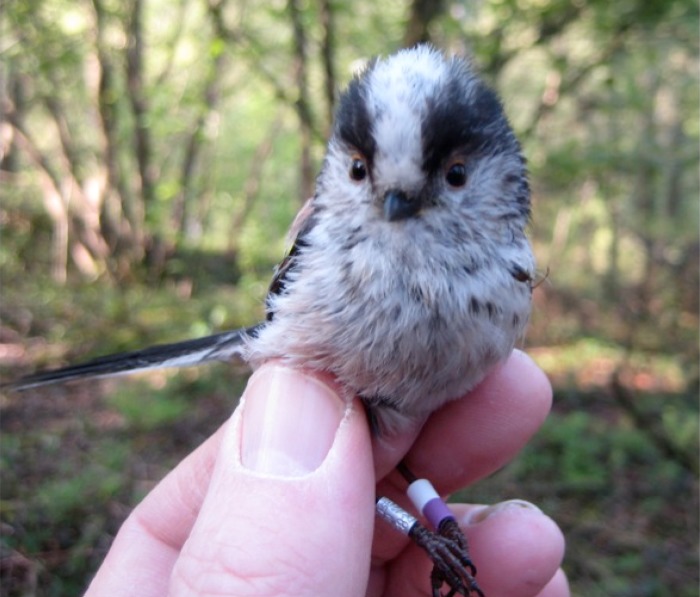
Adult long-tailed tit showing rings that allow individual recognition in the field. (Online version in colour.)

In this paper, we use data collected from a long-term field study of a cooperative breeder, the long-tailed tit (figure 1), to test Hamilton's rule. Long-tailed tits have several advantages over most cooperative species for this analysis, the most important being the relative simplicity of their cooperative breeding system, in which all helpers are failed breeders that redirect their care to help feed nestlings belonging to other pairs. Furthermore, they are very short lived compared with most cooperative species, facilitating measurement of the costs and benefits of alternative behaviours and allowing the rapid accumulation of complete life histories for the estimation of lifetime reproductive success [35]. We first estimate the parameters *r*, *b* and *c*, and then test whether Hamilton's condition for the evolution of apparently altruistic helping behaviour is satisfied in this species.

## 2. Study system

The cooperative breeding system of long-tailed tits has been described in detail elsewhere [39,40], so here we simply outline its main features. Long-tailed tits spend the non-breeding season in flocks that occupy large non-exclusive ranges that typically comprise 6–16 birds, including members of one or more nuclear families and a number of unrelated immigrants who disperse between flocks during the autumn and winter. In early spring, flocks start to break up and pairs form. There is an approximately equal adult sex ratio, and at the start of the breeding season all birds attempt to breed independently in socially monogamous pairs that occupy undefended, non-exclusive ranges. Each pair builds their own nest, an intricate domed structure that is normally sited in vegetation within 1–3 m of the ground. Clutch size is typically 9–11 eggs (mode = 10); the female incubates alone for 13–16 days, commencing incubation on the day of clutch completion. Chicks hatch synchronously and are fed in the nest for 16–17 days until fledging. Families may merge soon after fledging, and dispersal between flocks commences once juveniles are independent and continues throughout the non-breeding season.

Long-tailed tits are single-brooded, but they suffer a high nest-failure rate, caused mainly by predators (72% of all nests). Early in the season, failed breeders attempt to breed again, but if failure occurs after late April or early May, pairs abandon breeding for that year and a proportion of these failed breeders (especially males; 85% of all helpers) become helpers at the nest of another pair, assisting them by feeding their nestlings and fledglings [41]. As a result of the high nest-failure rate and resulting failed breeders, about half of all successful broods have helpers and helped nests have a mean of 1.8 helpers [42].

We have studied a population of long-tailed tits in the Rivelin Valley, Sheffield, UK (53°23′ N, 1°34′ W) since 1994. The population varies in size, but averages about 100 breeding adults during the breeding season, of which more than 95% are ringed each year with unique combinations of colour rings. We attempt to find all nests and closely monitor them, recording the timing of breeding, clutch size, hatch date (day 0), and the identity and provisioning rates of carers on alternate days from day 2 until fledging or nest failure. In the event of nest failure, we intensively search the study area for new attempts. Nestlings are ringed, weighed and measured on day 11 of the nestling period, and a small blood sample taken by brachial venipuncture (under UK Home Office Licence); blood samples are also taken from all adults at the time of first capture. DNA is extracted from blood samples and all sampled birds are genotyped at 19 microsatellite loci and sexed using two independent sex markers [43,44]. Importantly, the study has followed the same protocols since its inception, with a similar intensity of fieldwork in each year, with the exception of 2001, when access to the study site was severely constrained by restrictions imposed following an outbreak of foot-and-mouth disease; data from 2001 are therefore excluded from most analyses.

In our analyses, we consider the costs and benefits of helping for an average helper, regardless of their sex. However, to determine the effect of helpers on their own and recipients’ fitness, we estimate the marginal effect of an individual helper on the current and future productivity of male recruits, as previously [41]. We focus on male recruitment because most juvenile females disperse out of the study population in their first winter, while most males are philopatric, so the local recruitment rate of all fledglings from a given brood will be a function of brood sex ratio. By measuring the recruitment rate of male offspring only (determined genetically), we can be reasonably confident that we have detected all survivors, and we reduce the confounding effect of dispersal on survival estimates.

## 3. Components of Hamilton's rule

### (a) Relatedness

In previous studies of long-tailed tits [39,45] and from the early days of this study [46], it was apparent that helpers typically redirect their care towards relatives. This pattern does not emerge through random choice of beneficiaries in a strongly kin-structured population, but rather as a result of active kin discrimination. First, when offered a choice between helping at nests belonging to kin or non-kin, while controlling for spatial effects, failed breeders exhibit a strong preference for helping kin [21]. Second, there is a positive correlation between the rate at which helpers provision nestlings and their mean relatedness to the brood, indicating that helper provisioning rules permit adjustment of care with respect to kinship [23,44]. Kin recognition is achieved using vocal cues that are learned during development [47–49], but the mechanism through which different degrees of kinship are perceived and discriminated, allowing adjustment of care in relation to kinship, is unknown. In addition, it is clear that although long-tailed tits prefer to help kin, a substantial proportion of helpers care for non-kin [23].

**Figure 2.**
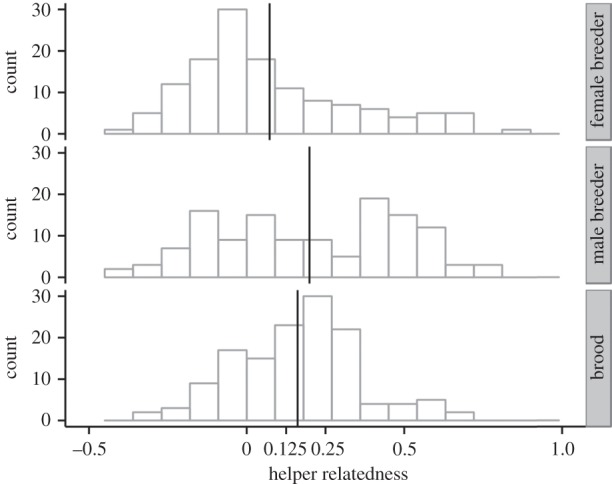
Frequency distributions of genetic relatedness of helpers to female breeder (*n* = 186), male breeder (*n* = 167) and brood (*n* = 186) in long-tailed tits. Individuals who were observed to provision a nest only once were excluded. Estimates were made using molecular markers (see [44] for details of genotyping methods).

Here, we have analysed the relatedness of helpers to male and female breeders and the helped brood using genetic data, the latter based on a set of 19 microsatellite markers that gives us better resolution of relatedness than previous genetic analyses [44]. We calculated pairwise relatedness from the markers using SPAGeDi 1.4 [50] with the estimator of Queller & Goodnight [51]. The distribution of relatedness for helpers who provisioned a brood at least twice is shown in figure 2 and is similar to that described by Nam *et al*. [23]. A substantial majority of helpers assist at a nest where they are typically related to one of the breeders, usually the male, but a substantial proportion help non-kin, whether assessed via social pedigree [23] or genetically (figure 2). The average relatedness *r* (±s.e.) of helpers to the male breeder whose brood they care for (*r_h−m_*) is 0.20 ± 0.02 (*n* = 167 helpers), to female breeders (*r_h−f_*) is 0.07 ± 0.02 (*n* = 186) and to broods (*r_h−b_*) it is 0.16 ± 0.01 (*n* = 186). Thus, as in many other studies [36], we have been able to measure a robust and consistent value of mean relatedness, *r*, between helpers and the beneficiaries of their help with a high degree of confidence.

### (b) Benefits of helping

Helpers may benefit the recipients of their care by increasing productivity of the current brood. This may be achieved by reducing the probability of nest predation [52] or by increasing the rate at which young are provisioned, resulting in increased survival at the nestling and/or post-fledging stage [53,54]. In addition, by assuming some of the burdens of parental care, helpers may reduce breeders’ costs of parental care (‘load-lightening’ [55]), thereby enhancing parents’ residual reproductive value. We consider each of them in turn.

#### (i) Production of related offspring

**Figure 3.**
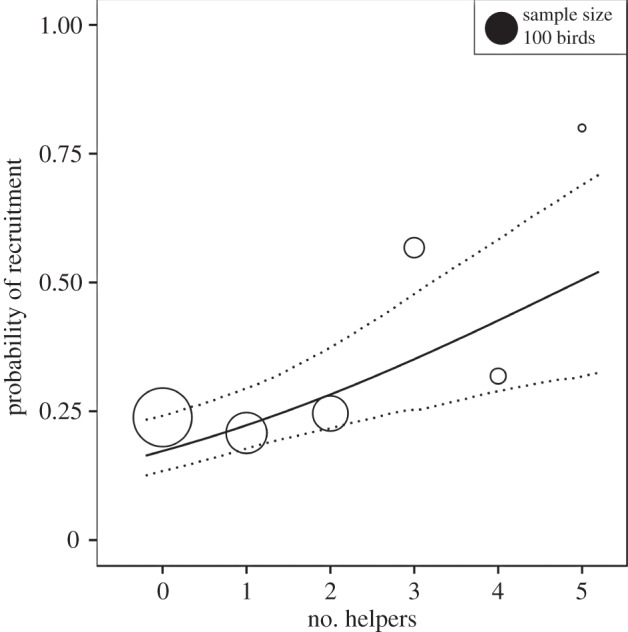
Probability of recruitment for male fledgling long-tailed tits in relation to the number of helpers that provisioned them as nestlings. Circles show the observed proportions of recruited males for each number of helpers, scaled by sample size. Solid line shows model fitted probability of recruitment for males from a multilevel logistic regression [58] on data for males and females (*n* = 1242; males = 672). The model had fixed effects for sex and number of helpers and random effects for year (1994–2009, excluding 2001) and nest ID (163 nests). The fixed part of the model was logit^−1^(−1.57–0.99 × female + 0.32 × helpers). Dotted lines show 95% confidence intervals calculated from simulations of the posterior distribution of each model parameter [59].

In contrast to typical cooperative breeding systems where helping occurs within discrete, stable and often long-lived groups, the cooperative association between breeders and helpers in long-tailed tits is relatively brief. In particular, the presence of a helper at the nest is unpredictable because becoming a helper depends initially on the stochastic event of nest predation, and helpers are very rarely associated with a nest before the nestling period [56]. As a consequence, helpers can directly influence productivity measures only in the latter stages of the nesting cycle. We have previously shown that the presence of helpers has no significant positive effect on the probability of total brood failure through predation because long-tailed tits are ineffectual at deterring predators such as weasels *Mustela nivalis* and jays *Garrulus glandarius*. There is also a low rate of nestling starvation (less than 3% between hatching and ringing on day 11), so there is little opportunity for helpers to influence brood size at this age, nor evidence that they do so [56]. However, despite the fact that parents reduce their work rate when they have helpers (see §3*b*(ii)), the contribution of helpers increases the total rate at which broods are provisioned [57], and hence nestling condition at day 11 increases with the number of helpers at the nest [56]. This effect of helpers on nestling condition has long-term consequences for offspring fitness because the probability of a fledgling recruiting into the breeding population as a 1-year old also increases with the number of helpers that fed them (figure 3). We cannot exclude the possibility that this effect is influenced by post-independence events arising from continuing association with helpers through the non-breeding season. However, we consider this unlikely because the fluid nature of post-breeding flocks of long-tailed tits means that discrete family plus helper groups are soon disrupted by natal dispersal and amalgamation of flocks [60].

Importantly, the effect of helpers on recruitment of helped broods is fairly linear, at least within the natural range of helper numbers that we have observed (figure 3). This facilitates estimation of the marginal effect of helpers on productivity because there is little dependence on group size. From the model in figure 3 and the derivatives of the logistic function, the marginal effect of an additional helper on the recruitment of an individual male fledgling was +6.2% (95% confidence interval (CI) = 2.4–11%). With mean brood size of 8.9 fledglings [56] and brood sex ratio of 0.53 male [61], we estimate the mean marginal effect of a helper on recruitment as +0.292 male recruits (i.e. 8.9 × 0.53 × 0.062) (95% CI = 0.11, 0.53).

#### (ii) Survival of related breeders

We have abundant observational and experimental evidence that the care of long-tailed tit helpers is not simply additive to that provided by parents but also allows breeders to reduce the rate at which they provision their own chicks, thereby potentially reducing their parental care costs [44,46,56,57]. Meade *et al*. [62] investigated the extent of this load-lightening effect on male and female breeders and its consequences for their subsequent survival and fecundity using data collected over 14 years. Both sexes provisioned their broods at lower rates when helped, but load-lightening was asymmetric, with females reducing their effort more when feeding small broods and males reducing their effort more at the larger brood sizes that are typical of the species. Thus, males made the biggest marginal reduction in their provisioning rate when helped, and this differential reduction in provisioning effort in the presence of helpers according to sex was reflected in higher survival of males when caring for large broods, with no such increase in survival for females. At the modal brood size of nine nestlings in our study population [56,62], the effect of this load-lightening effect is to increase a male's probability of survival to the following breeding season from 0.43 without helpers to 0.52 with helpers, i.e. a marginal effect on male survival probability of +0.09 (fig. 3 in Meade *et al*. [62]).

The effect of helpers on male breeders’ reproductive success was estimated as follows. Given that the mean number of helpers at helped nests is 1.8 [42], the marginal effect of one helper on male breeders’ annual survival is +0.09/1.8 = +0.05. The mean annual probability that a pair produces a male recruit is 0.26 ± 0.20 s.d. (*n* = 18 years, range 0.03–0.77; P. R. Gullett 2014, unpublished data), so the net effect of a helper on a male breeder's production of male recruits in the following year is +0.05 × 0.26 = +0.013 male recruits. However, we must also take into account the relatedness of the male breeder to his own future brood, which is assumed to be 0.48, i.e. allowing for the low rate of extra-pair paternity [63]. Therefore, the marginal effect on a male breeder's future reproductive success per helper is estimated to be +0.013 × 0.48 = +0.0062 male genetic equivalents.

In these analyses, we have considered the effects of helpers in year *n* on recipients up to year *n* + 1, but not beyond. This is for two reasons. First, long-tailed tits are short lived so relatively few birds survive beyond 2 or 3 years. Second, we have no evidence for any longer term effect of helpers on recipients: helpers have no effect on the survival of helped offspring beyond the first year of life [64], and having helpers in year *n* does not influence recipients’ timing of breeding, clutch size or probability of breeding successfully in year *n* + 1 nor their probability of producing recruits in year *n* + 2 [65]. Therefore, we think it is reasonable to consider the benefits of helping for just 1 year after the helping event.

### (c) Costs of helping

There are several potential costs of helping in typical cooperative breeding systems [66]. First, helping is usually defined explicitly by the provision of alloparental care [67,68], and one of the fundamental tenets of life-history theory is that reproductive investment is costly [69,70]. Thus, all else being equal helpers are expected to incur some survival cost from their investment in broods. Evidence for such costs is widely assumed, but there are few good empirical examples [66,71,72]. Second, helpers may incur opportunity costs if helping occurs at the expense of breeding. Independent breeding is often not a realistic option for helpers because they are constrained from taking up reproductive opportunities by a shortage of territories, mates or other key resources [8] or because they live in family groups so that breeding would entail inbreeding [73]. Nevertheless, if helping and breeding are mutually exclusive activities, some opportunity cost of helping is likely. Of course, the various alternative options faced by an individual at the point it decides to help may be hard to define, so this cost of helping may be very hard to determine in many instances. Finally, there may be a cost associated with raising potential future competitors for territories, mates or dominance [74–76].

Here, we determine the survival costs of providing alloparental care and the opportunity cost of helping in long-tailed tits. Any future costs of competition with the helped brood for territory, mates or other resources are likely to be very small or negligible because long-tailed tits are not territorial [60], virtually all birds are able to find mates and attempt to breed each year [56], and adult survivorship is only very weakly density dependent with respect to total population size [77]. This absence of strong density dependence in survival is unsurprising in social species where Allee effects are likely to operate [78]. In the specific case of long-tailed tits, there are known benefits of communal roosting [79] as well as potential anti-predator benefits from social foraging that may mitigate any density-dependent processes influencing survival.

#### (i) Survival cost of helping

In principle, comparison of the survival rate of failed breeders that help with that of failed breeders that do not help would reveal any survival cost of helping, all else being equal. However, counterintuitively, such comparisons suggest that rather than being costly, helping confers survival benefits, with failed breeders that become helpers having significantly better survival rates than failed breeders that do not become helpers [64,65]. However, Meade & Hatchwell [65] concluded that helpers gain no direct fitness benefits from their helping behaviour, either through improved survival or future fecundity. Instead, this apparent survival advantage arises because all is not equal between the two categories of failed breeders; rather, there are quality differences among them that probably cause these survival differences. The key finding was that 42% of failed breeders with the opportunity to help (i.e. those that had a close relative with an active nest in the population) spurned the opportunity to help. These birds appeared to be of relatively low quality because they initiated their own breeding attempts later than those birds that did become helpers, and because they had a much lower survival rate to the following breeding season (0.24) than those birds that did choose to become helpers (0.56). Therefore, we think that the self-selected category of birds that decide to help are in better condition or of higher quality than the category of birds that do not help despite having the opportunity to do so. The third category of failed breeders did not have an opportunity to help because they had no relative with an active nest in the population at the time their own breeding attempt failed and these birds had a survival rate of 0.56 (i.e. the same as failed breeders that helped). If we assume that this category of birds included individuals with a similar distribution of condition/quality as those birds that had the opportunity to help, then we can estimate that they comprised 42% poor-quality birds with a survival rate of 0.24 and 58% high-quality birds with a survival rate of 0.79 (overall mean survival = 0.56). We then used the difference between estimated survival of good-quality birds that did help (0.56) and did not help (0.79) to estimate that investment of alloparental care by helpers has a survival cost of −0.23.

**Figure 4.**
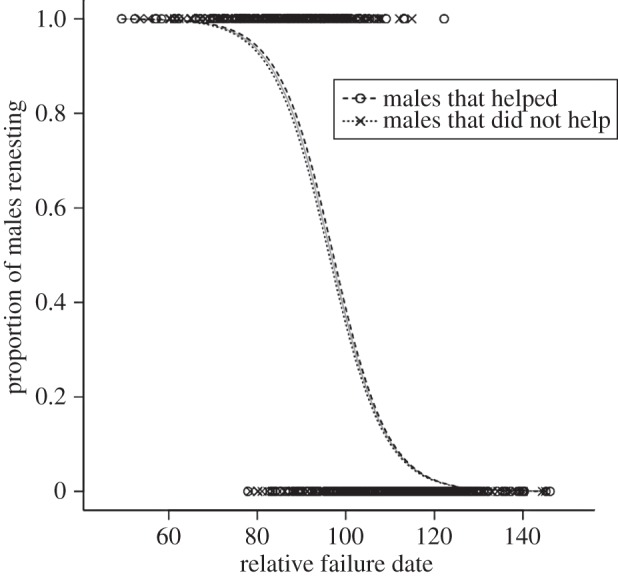
The probability of renesting following the failure of a breeding attempt in relation to standardized date of the breeding season for males that became helpers and males that did not become helpers. Failure dates and helping/renesting behaviour of breeders were recorded in the Rivelin Valley population over 18 years (1995–2013, with 2001 excluded). We excluded pairs that failed before the median lay date of first attempts in that year. In each year, we estimated ‘termination date’ as the date when 50% of females were expected to give up breeding, by modelling female renesting versus termination as a function of failure date in a series of annual logistic regressions (see [80] for full methods). We then calculated ‘relative failure date’ of breeding males as absolute failure date minus termination date in that year, plus 100 to remove negative values. This ‘relative failure date’ was calculated for all breeding males with known ID (*n* = 377). We split the 868 nests belonging to these males into two subsets depending on their behaviour following nest failure: those that eventually went on to help at a nest of another bird in that year (*n* = 413), and those that did not help after failing but instead terminated breeding for that year (*n* = 455). We then modelled the probability of males renesting versus terminating breeding, in response to relative failure date, using a binomial model with logit link function. We repeated this for helpers and non-helpers separately. The overall mean inflection point (50% probability of renesting) was 96.7 days ± 0.65 s.e. and did not differ for males that became helpers (97.2 ± 0.93 s.e.) and non-helpers (96.2 ± 0.91 s.e.).

This estimated survival cost of helping may sound surprisingly high, but it is consistent with the effect of load-lightening for male breeders described above. A male helper that feeds throughout the nestling period contributes 2.45 times the number of feeds that a male breeder saves through load-lightening if helped throughout the nestling period (calculated from figure 4 in MacColl & Hatchwell [57]). The ratio of helper survival cost (0.23) to parental survival benefit (0.09) is remarkably similar at 2.56, indicating that these survival consequences of helping and load-lightening are appropriately scaled.

Following the same rationale that we used to convert breeder survival benefits into future productivity, i.e. mean annual probability of producing male recruits of 0.26, the marginal effect of helping on production of future male recruits is −0.23 × 0.26 = −0.0598 male recruits. Again, assuming that relatedness of a male breeder to the young in his nest is 0.48 we estimate the marginal cost of helping via reduced helper survival as −0.0598 × 0.48 = −0.0287 genetic equivalents.

#### (ii) Opportunity cost of helping

Almost all helpers in our study population are failed breeders that redirect their care [56]. In some years, one or more previously unknown birds appear at nests, often close to study site boundaries, but these birds are likely to be failed breeders from outside the study site that have moved to become helpers. Thus, we have no evidence that long-tailed tits in our population forego breeding altogether to become helpers, implying that there is no opportunity cost of helping. However, even if all birds do initially breed each year there may still potentially be a more subtle opportunity cost of helping if failed breeders that become helpers are less likely to attempt to re-nest than failed breeders that did not have that opportunity. We have previously shown that the decision to terminate breeding in a given year occurs within a narrow time window at the end of a temporally constrained breeding season [41,56,80]. Here, we examined whether the transition from breeding to not breeding occurred earlier for birds that became helpers than for those that did not, effectively resulting in missed opportunities for independent breeding.

Using data collected over 18 years, we tested whether males that became helpers abandoned breeding following nest failure earlier than males that did not become helpers (analysis was restricted to males because most helpers are male). The end of the breeding season varies between years according to April temperature [80], so we analysed renesting decisions relative to a standardized breeding termination date for ease of comparison across years. The dataset comprised information for 377 males involved in 868 breeding attempts. We found that there was almost perfect synchrony in the decision to abandon breeding by failed breeders that became helpers and those that became non-helpers (figure 4), demonstrating that long-tailed tit helpers suffer no cost of lost opportunities for independent breeding.

## 4. Testing Hamilton's rule: is *rb* > *c*?

The terms required to parametrize Hamilton's rule are summarized in table 1. The first thing to note is that we have shown that helpers incur a fitness cost via decreased survival that translates to a loss of 0.0287 genetic equivalents. Therefore, helping reduces the individual's direct fitness and can be described as altruistic. However, when we parametrize Hamilton's inequality as follows:

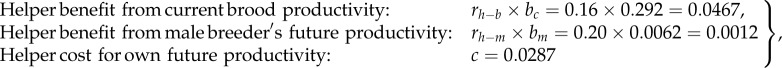

**Table 1.** Summary of the values and calculations used to generate the terms of Hamilton's rule, *r*, *b* and *c*, in long-tailed tits. Values in bold are those used in parametrizing Hamilton's rule.

term	marginal effect of helper on current brood productivity	marginal effect of helper on future productivity of male breeder	marginal effect of helping on helper's future productivity
*r*	relatedness of helper to brood, →*r_h−b_* = **0.16**	relatedness of helper to helped male, →*r_h−m_* = **0.20**	relatedness of helper to self, *r_h−h_* = 1
*b*	brood size = 8.9proportion of brood male = 0.53 Δ recruitment rate = +0.062 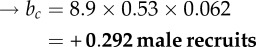	Δ survival rate = +0.05 probability of producing male recruit in year *n* + 1 = 0.26 →0.05 × 0.26 = +0.013 male recruits relatedness of breeder to recruits = 0.48 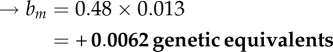	none
*c*	none	none	Δ survival rate = −0.23 probability of producing male recruit in year *n* + 1 = 0.26 →−0.23 × 0.26 = −0.0598 male recruits relatedness of helper to recruits = 0.48 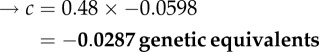

we find that Hamilton's rule is satisfied because when weighted by relatedness, the combined helper benefit via the current brood (0.0467) and male breeders (0.0012) of 0.0479 genetic equivalents is greater than the cost of 0.0287 genetic equivalents. In other words, the benefits of helping exceed the costs by approximately 67%, the great majority of the benefit being derived from the effect of helpers on offspring recruitment from the helped brood.

## 5. Implications and conclusion

Our results indicate that helping in long-tailed tits is altruistic at the level of the individual because helpers incur a direct fitness cost of reduced survival by helping. Nevertheless, despite the low average relatedness between helpers and the recipients of their care in this species, helpers derive indirect fitness benefits by increasing the number of recruits from helped broods and increasing the survival of breeders. Overall, these effects satisfy Hamilton's rule for the evolution of altruistic behaviour. We first discuss the relative importance of direct and indirect benefits of helping in this species, and then consider the broader implications for studies of other cooperative breeders and the significance of using inclusive fitness estimates over alternative fitness metrics.

Among previous estimates of the index of kin selection [27], just two studies (white-fronted bee-eaters *Merops bullockoides* [37] and pied kingfisher *Ceryle rudis* primary helpers [81]) have quantitatively concluded that helping is altruistic, i.e. that helpers decrease their own future probability of helping and that helping persists only because of indirect fitness benefits. In other cases, it was concluded that helpers gained both direct and indirect fitness benefits [31]. Of course, the *b* in Hamilton's rule is the effect of actors only on the fitness of recipients and does not include any direct fitness benefit that an altruistic actor might accrue. In the context of cooperative breeding, such benefits might include access to parentage in current brood, access to enhanced group benefits, prolonged parental care and acquisition of skills that increase personal survival and future reproductive success [10,82]. These direct fitness benefits of helping may be substantial and outweigh any costs of helping, resulting in selection for helping behaviour even in the absence of any kin-selected benefits. Therefore, it is important to note that we have found no evidence that helpers accrue any direct fitness benefit in terms of current reproduction in the brood being helped [63], or increased personal survival or future reproduction in later breeding attempts [65]. This allows us to draw the general conclusion that by showing that Hamilton's rule is satisfied, we have effectively demonstrated that helping behaviour in long-tailed tits must be the product of kin selection, a point that we have previously concluded [40] but not shown through the calculation of the terms of Hamilton's rule.

Another important conclusion is that although helping in long-tailed tits is kin-selected, this does not mean that all helpers must be related to the recipients of their care (figure 2). Indeed, it is interesting that mean relatedness of helpers to broods in long-tailed tits (0.16) is substantially lower than is commonly observed in other cooperatively breeding species, where help is often directed primarily towards full- and half-siblings [6]. It is frequently suggested that care for non-kin by helpers allows kin selection to be discounted as an explanation for cooperative breeding. This is clearly not the case, and as argued by Nam *et al*. [23] and made explicit here, altruistic care for non-kin by a fraction of helpers may occur even if there is no benefit accrued by these helpers. The proportion of helpers that might be expected to help in the absence of any direct or indirect fitness benefits will be a function of the relative costs and benefits of helping, and the selection these exert on the discrimination rules used when making helping decisions [26]. These considerations are likely to be species specific and result in help for non-kin being rare in some species but frequent in others.

Finally, we consider the significance of using inclusive fitness estimates when studying the evolution of social traits in this species. In practice, classical fitness [83] or neighbour-modulated fitness [1] estimates may be relatively easily derived empirically for individuals by recording, for example, the total number of offspring produced, or the number of recruits into the adult population or even the number of grand-offspring attributable to each individual. Fisher [83], cited in Foster [84], regarded the potential indirect effects of an individual on its relatives’ fitness to be generally unimportant compared to personal reproduction. Hamilton [1] was more concerned about the scale and attribution of any indirect effects and was explicit in his definitions of neighbour-modulated and inclusive fitness. A critical point we make here is that although the mean neighbour-modulated and inclusive fitness measured for a given population should be the same, the distribution of fitness among individuals may differ radically. MacColl & Hatchwell [35] used lifetime reproductive success data from long-tailed tits to determine fitness (production of recruits to the breeding population), partitioning inclusive fitness into its direct and indirect components. A principal finding was that just 31% (70/228) of birds that survived to breed achieved any inclusive fitness, and, of those 70 individuals, 21% (15/70) achieved no direct fitness (or neighbour-modulated fitness) and gained fitness only indirectly via helping. Thus, the variance in fitness among individuals was substantially lower when estimating inclusive fitness compared to using neighbour-modulated fitness. This is very likely to be a general finding in cooperative breeding systems because more individuals usually have the opportunity to help than have the opportunity to breed. We are currently exploring in more detail the implications of using alternative metrics of fitness for our understanding of selection on behavioural (e.g. social) and life-history traits.

In summary, using robust measures of relatedness, benefits and costs from a long-term study, we have shown that long-tailed tit helpers are altruistic because they incur direct fitness costs from their cooperative behaviour. However, these costs are outweighed by gains in indirect fitness, principally through the increased recruitment of related offspring from helped broods. Therefore, helping behaviour in this species is consistent with Hamilton's rule. This is despite the fact that relatedness between helpers and the recipients of their care is, on average, low relative to many other cooperatively breeding species. Indeed, the relatively high benefits and low costs of helping in long-tailed tits have selected for decision rules that result in a substantial proportion of helpers caring for non-kin even though they gain neither direct nor indirect fitness benefits from doing so. Thus, we conclude that even though, on average, Hamilton's rule is satisfied, apparently maladaptive help for non-kin occurs in long-tailed tits and would be expected to occur in other kin-selected cooperative breeding systems.
